# Distantly related lipocalins share two conserved clusters of hydrophobic residues: use in homology modeling

**DOI:** 10.1186/1472-6807-8-1

**Published:** 2008-01-11

**Authors:** Benoit Adam, Benoit Charloteaux, Jerome Beaufays, Luc Vanhamme, Edmond Godfroid, Robert Brasseur, Laurence Lins

**Affiliations:** 1 Centre de Biophysique Moléculaire et Numérique, Faculté Universitaire des Sciences Agronomiques de Gembloux, Gembloux, Belgium; 2 Service de Génétique Appliquée, Institut de Biologie et de Médecine Moléculaires, Université Libre de Bruxelles, Gosselies, Belgium; 3 Laboratoire de Parasitologie moléculaire, Institut de Biologie et de Médecine Moléculaires, Université Libre de Bruxelles, Gosselies, Belgium

## Abstract

**Background:**

Lipocalins are widely distributed in nature and are found in bacteria, plants, arthropoda and vertebra. In hematophagous arthropods, they are implicated in the successful accomplishment of the blood meal, interfering with platelet aggregation, blood coagulation and inflammation and in the transmission of disease parasites such as *Trypanosoma cruzi *and *Borrelia burgdorferi*.

The pairwise sequence identity is low among this family, often below 30%, despite a well conserved tertiary structure. Under the 30% identity threshold, alignment methods do not correctly assign and align proteins. The only safe way to assign a sequence to that family is by experimental determination. However, these procedures are long and costly and cannot always be applied. A way to circumvent the experimental approach is sequence and structure analyze. To further help in that task, the residues implicated in the stabilisation of the lipocalin fold were determined. This was done by analyzing the conserved interactions for ten lipocalins having a maximum pairwise identity of 28% and various functions.

**Results:**

It was determined that two hydrophobic clusters of residues are conserved by analysing the ten lipocalin structures and sequences. One cluster is internal to the barrel, involving all strands and the 3_10 _helix. The other is external, involving four strands and the helix lying parallel to the barrel surface. These clusters are also present in RaHBP2, a unusual "outlier" lipocalin from tick *Rhipicephalus appendiculatus*. This information was used to assess assignment of LIR2 a protein from *Ixodes ricinus *and to build a 3D model that helps to predict function. FTIR data support the lipocalin fold for this protein.

**Conclusion:**

By sequence and structural analyzes, two conserved clusters of hydrophobic residues in interactions have been identified in lipocalins. Since the residues implicated are not conserved for function, they should provide the minimal subset necessary to confer the lipocalin fold. This information has been used to assign LIR2 to lipocalins and to investigate its structure/function relationship. This study could be applied to other protein families with low pairwise similarity, such as the structurally related fatty acid binding proteins or avidins.

## Background

Lipocalins are small secreted proteins (160–200 residues), typically structured in a 8 strands up and down β-barrel. A 3_10 _helix closes one extremity of the barrel (H1) and a second is found parallel to its surface (H2). The interior of the cavity can hold a small, typically hydrophobic, molecule. Each lipocalin is usually well adapted to the recognition of its ligand. Lipocalins can also bind to receptors and be part of macromolecular complexes. They are involved in numerous functions such as in the transport of molecules implicated in homeostasis (e.g. retinoids, arachidonic acid), enzymatic synthesis, immunomodulation, olfaction, pheromone signaling and cell regulation [1]. The sequence identity is low among this family despite a well conserved tertiary structure. For lipocalins with differing biochemical functions pairwise identity can fall below 10% [2]. However, there is a core set of lipocalins, called 'kernel', that are quite closely related proteins. They share three structurally conserved regions (SCRs). The more divergent lipocalins, called outliers, match no more than two of the SCRs [3].

Recognition of the SCRs permits assignment to the lipocalin family membership. However, for members not sharing the SCRs motifs, structural determination is the only safe way to decide their relationship to the family. Another strategy to decide their assignment is through the analysis of their exon-intron structure [4,5]. For instance, RaHBP2 was assigned to the lipocalin family only by its structural properties [6]. RaHBP2 is a histamine-binding lipocalin from the hard tick *Rhipicephalus appendiculatus *with two binding pockets. The pocket at the bottom of the barrel is the low affinity binding site (L) and contains two negatively charged residues. The one near the mouth is the high affinity binding site (H) and contains four negatively charged residues. Its similarity with other members of the lipocalin family is very low. Furthermore it has an α-helix instead of a 3_10 _helical structure closing the barrel.

Lipocalins are widespread across species and are found in various organisms such as bacteria, plants, arthropoda and vertebra [1]. Up to now, they have not been evidenced in the Archaea domain, but this might be due to the fact that it is difficult to identify lipocalins not sharing the SCRs. Otherwise, an increasing number of sequences with an identity around 15% with lipocalins and missing lipocalin recognition motifs are found in protein databanks. In blood sucking arthropods, many lipocalin-related sequences, expressed in the salivary glands, have been identified [7]. Several have been characterized, notably RaHBP2, and were found to be implicated in the completion of the blood meal, interfering with platelet aggregation, blood coagulation, activation of the complement system and inflammation. They are also implicated in the transmission of disease parasites such as *Trypanosoma cruzi *and *Borrelia burgdorferi *and tick toxicoses [8-10]. However, most of the expressed sequences, among them LIR 2 from tick *Ixodes ricinus*, have unknown functions and have a pairwise sequence identity with experimentally identified lipocalins within or below the twilight zone [9,11,12].

The question is how to confirm that they belong to the lipocalin family and determine their function without solving their structures, which could be a long and difficult process. Homology modeling has up to now been the only method available to predict the 3D structure of proteins of this size, with an accuracy comparable to a low-resolution experimental structure [13]. Prediction of a 3D model by homology modeling requires 30% identity. It has been determined that above a cut-off of 30% sequence identity, 90% of the pairs are homologous and have an equivalent structure; below 25%, less than 10% are [12]. This implies that under this limit, the correct assignment of an homologous template becomes less reliable, as well as the alignment between the target and template sequences. Sequence identity between lipocalins is far under this limit. It should be noted that this is not an exception since Rost has determined that most of the similar protein structure pairs in the PDB appear to have less than 12% pairwise sequence identity [14]. Therefore, before considering the construction of a 3D model with a low level of identity between the template and target, the validity of the template must be confirmed and the alignment optimized. This can be done by comparing predictions of secondary structures and accessibility to the solvent, patterns of hydrophobic and peculiar residues.

Proteins can share a similar 3D-structure with low sequence similarity only if the fold is not determined by all details of the sequence but by key features [15,16]. When comparing the structures of proteins with low similarity, it is usual that a set of clustered residues remains conserved; the latter form the structural core. Clarke et al. have identified a structural core for the immunoglobulin-like beta-sandwich proteins [16], Ptitsyn, a structural core for c-type cytochromes [17] and Socolich et al., a cluster of evolutionarily linked residues for the WW fold [18]. The structural core of lipocalins has not yet been analyzed taking into account the more distant outlier lipocalins [19,20].

The aim of this study is to provide arguments for the assignment of outlier lipocalins to this family and to help their alignment with a template for homology modeling. To achieve this goal, the conserved properties for lipocalins were identified and their structural core was analyzed using a set of ten structurally aligned lipocalins. These proteins have a maximum sequence identity of 28% and diverse functions. Due to the diversity of functions, it is likely that the characteristics identified as conserved could be important for the fold and not for the function. To identify the residues implicated in the structural core, the interactions conserved for these lipocalins were determined.

The results were thereafter used to confirm the assignment of LIR2, a tick protein, to the lipocalin family and to align it with RaHBP2. The latter is an atypical lipocalin since it does not share the SCRs and has an α-helix closing the bottom of the barrel. Nevertheless, it shares the main structural properties identified here for the lipocalins. The alignment was used to build a 3D model for LIR2. Despite the low sequence similarity between LIR2 and its template, the model enables hypotheses about its binding to histamine to be made, and experimentally validated.

## Results

### Study of the lipocalin family

**Figure 1:**
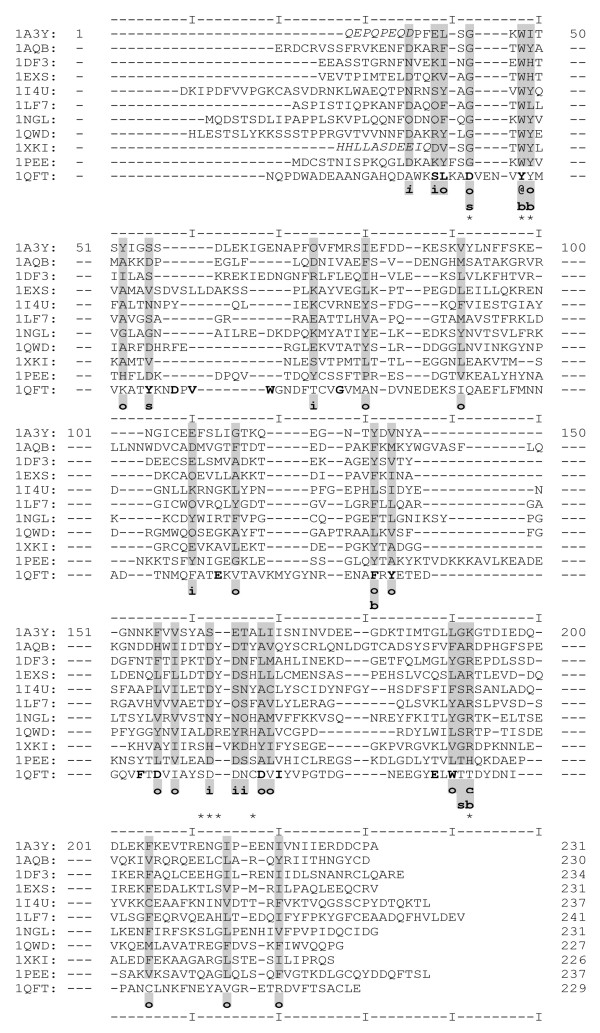
**Structural alignment obtained with VAST**. Positions with a conserved nature [i.e. hydrophilic (i), hydrophobic (o), aromatic (@), charged (c), bulky (b) and small (s)] for 10 lipocalins (1QFT not included) are highlighted in gray. Residues in italic are not present in the X-ray structure. Positions corresponding to SCRs as described by Flower and Col. [1] are indicated with a "*". Residues belonging to the active site of 1QFT are bold.

The lipocalins from our database (see methods) were structurally aligned with VAST. A gap in H1 for 1PEE was suppressed and another was introduced before the second helix of 1QFT to improve the structural correspondence. The structural alignment is presented in Figure 1 and the identity between the sequences in the table provided as supplementary material (Additional file 1). The identity varies from 5% to 28% with an average value around 15%, below the nominal threshold for a reliable sequence alignment [21]. Detection of residues in interaction in the structures was done using the PEX software [22]. Interactions are classified depending on the nature of the amino acids implicated, that is to say hydrophobic (Ala, Cys, Phe, Gly, His, Ile, Leu, Met, Val, Trp, Tyr, Pro), hydrophilic (Asp, Glu, Arg, Lys, His, Asn, Gln, Ser, Thr, Tyr), charged (Asp, Glu, Arg, Lys, His) and aromatic (Phe, His, Tyr, Trp). A maximum of 15 interactions were taken into account for each residue, because no residue has more than 15 interactions. An interaction is considered to be fully conserved when it is conserved for each structure and when the interacting residues are of the same nature (i.e. : hydrophobic, hydrophilic, charged or aromatic) throughout the alignment. If the interaction is not of the same type for one protein, it is considered to be not strictly conserved. For some structures of lipocalins (1XKI and 1A3Y) the H1 is not well resolved. The conservation of the barrel and H2 is thus considered separately from that of H1. Furthermore, as RaHBP2 (1QFT) is a peculiar lipocalin having an α-helix instead of a 3_10 _structure closing the barrel, and does not present the structurally conserved regions (SCRs) of typical lipocalins, it has been considered separately in the conservation analysis.

Prior to the interaction study, the conservation of the nature of residues (i.e.: hydrophobic, hydrophilic, aromatic, charged, see above for definition) was analyzed in the alignment. The size of the residue was also taken into account : Glu, Phe, His, Lys, Ile, Leu, Met, Gln, Arg, Trp, Tyr are considered as bulky and the others as small residues.

### Barrel and helix 2

#### Conservation in the alignment

The positions in the alignment for which a property is conserved for all 10 sequences are indicated in Figure 1. Among the 25 conserved positions, 6 are of a conserved hydrophilic nature, 16 hydrophobic, one is aromatic (Trp from SCR1) and one is charged (the negative residue in SCR3). Seven positions are conserved in size. Four are conserved as bulky residues and three as small, among which the conserved Gly of SCR1. Table summarizes the conserved positions in all the lipocalins. Less than a half of the conserved positions have their side chains external to the barrel (49, 52, 74, 110, 162, 165, 166, 169, 205, 214, 220). All positions conserved as hydrophobic, except residue 220, have a accessible surface area inferior to 30%. For residue 220, the accessibility is not conserved in 1XKI. Owing to the absence of the C-ter region and consequently the disulfide bond, residue 220 is more exposed to the solvent for that particular structure. The conserved hydrophilic positions do not show conservation for their accessibility to the solvent.

For 1QFT, positions 52, 55, 110, 156, 168, 192 and 220 are not conserved. Furthermore, position 42 (Asp) is not a Gly and 48 (Tyr) not a Trp. Noticeably, for all lipocalins and 1QFT, position 158 is conserved as a branched aliphatic residue (Ile, Val or Leu).

#### Conserved interactions

**Figure 2:**
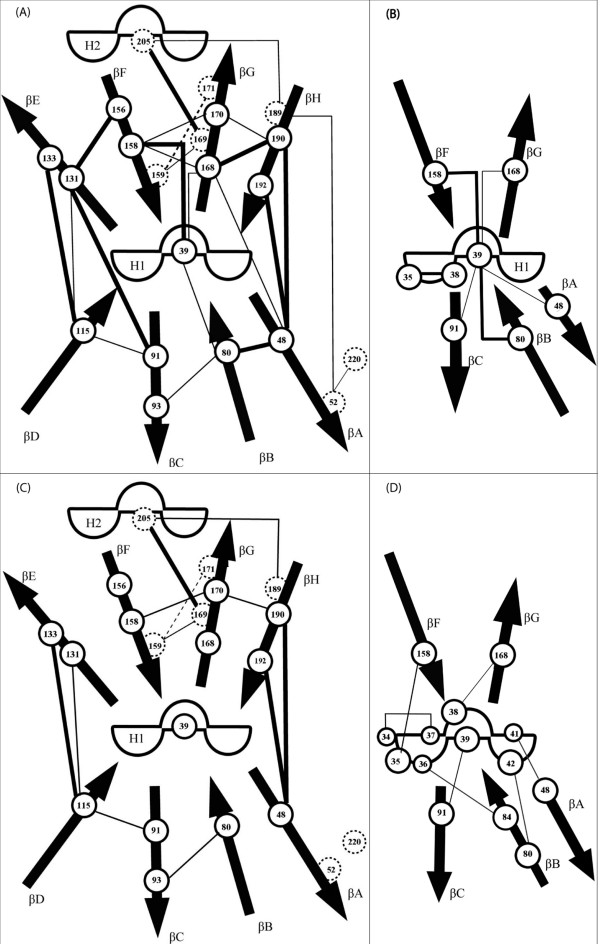
**Schematic representation of the interactions conserved in lipocalins**. The arrows represent the β-strands and the hemi-circles the helical structures. Positions belonging to the external cluster are represented by hatched circles. Interactions not strictly conserved are dashed. A. interactions conserved for 10/10 (bold) and 9/10 lipocalins (thin). B. interactions of H1 conserved for 8/8 (bold) and 7/8 (thin) lipocalins. C. interactions conserved for 11/11 (bold) and for both 9/10 lipocalins and 1QFT (thin). D. interactions of H1 of 1QFT.

**Figure 3:**
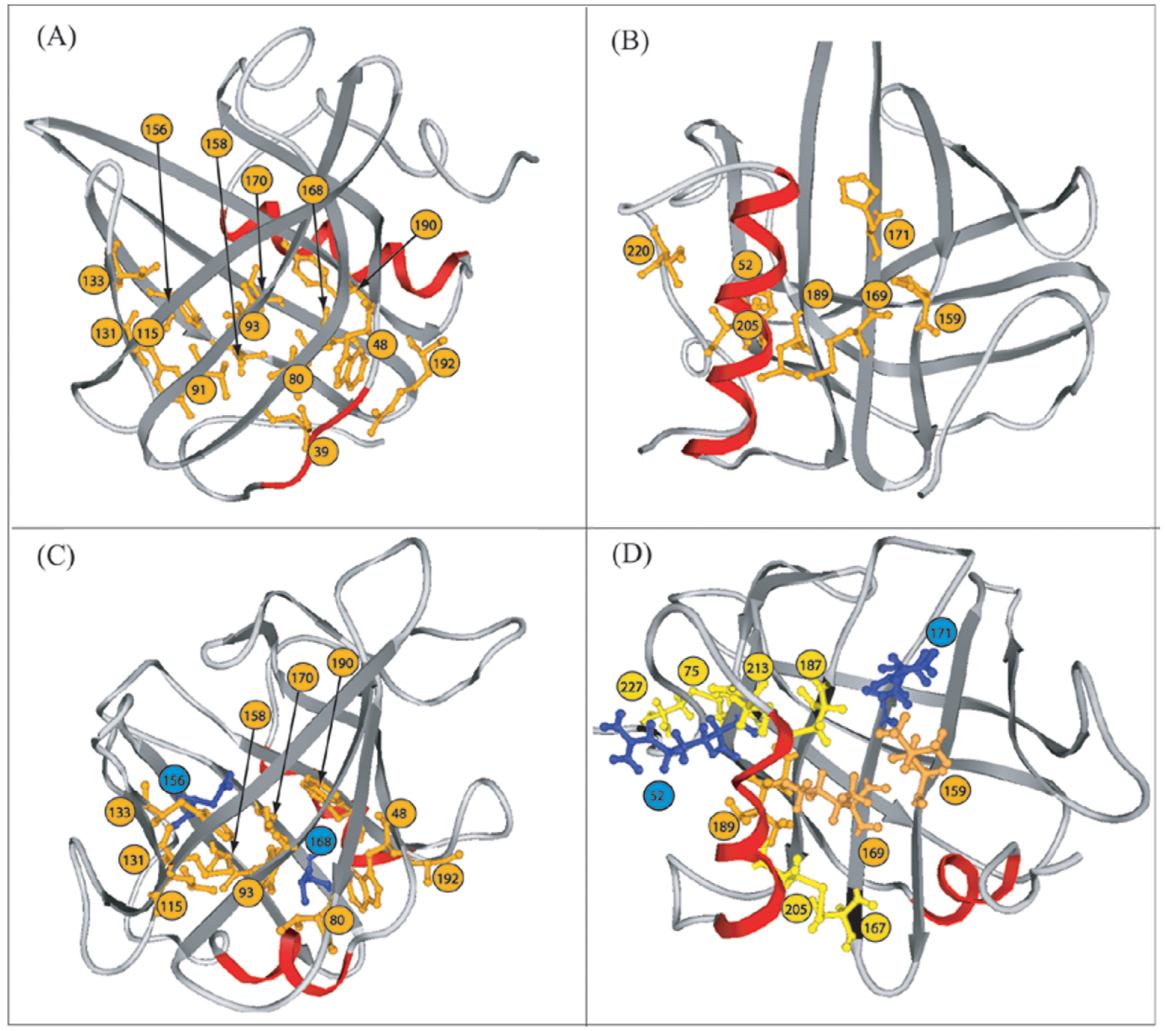
**Schematic ribbon representation of the structures of 1DF3 (A, B) and LIR2 (C, D)**. Residues implicated in the external (B, D) and internal (A, C) clusters are shown in ball and stick. Hydrophobic residues are represented in orange. For LIR2 the disulfide bonds are in yellow and the hydrophilic residues in blue. The hydrophobic belt includes positions (in brackets : corresponding residue for LIR2) 48 (Trp), 80 (Val), 91 (Phe), 93 (Tyr), 115 (Leu), 131 (Met), 133 (Phe), 156 (Asn), 158 (Val), 168 (Thr), 170 (Leu), 190 (Trp), 192 (Ala) and the external cluster 52 (Arg), 159 (Leu), 169 (Ile), 171 (Arg), 189 (Leu), 205(Cys), 220 (Phe; not interacting with the cluster).

The interactions conserved throughout the 10 structures (10/10) have been studied and are represented in Figure 2 and summarized in Table 1. All conserved interactions involve hydrophobic residues located on the interior of the barrel, except for that between 48 and 192 implicating a Trp and a basic residue (but interacting through their hydrophobic regions) and the 169–205 interaction located at the interface between H2 and the barrel (Figure 3A and 3B). The conserved interactions involve all strands of the barrel as well as the two helices.

**Table 1:** Conserved properties and interactions for barrel and helix 2.

Position	Struct. Info.		Conserved properties(10/10)			Conserved Interactions		
	In/out	2^nd^				11/11	10/10	9/10

42	in	loop	pho	**Gly**	Small (x)			
48	in	βA	pho (x)	arom (x)	bulky (x)		80	80
				**Trp**				168
						190	190	190 (x)
						192	192	192 (x)
49	out	βA	pho (x)		bulky (x)			
52	out	βA	pho					189
								220
55	in	Ω loop			small			
74	out	Ω loop	Phi (x)					
80	in	βB	pho (x)					39
							48	48
								93 (x)
91	in	βC	pho (x)					115 (x)
							131	131
93								80 (x)
110	out	βD	Phi					
115	in	βD	pho (x)					91 (x)
								131 (x)
							133	133 (x)
131	in	βE	pho (x)		bulky (x)		91	91
								115 (x)
							156	156
133	in	βE	pho (x)				115	115 (x)
156	in	βF	pho				131	131
158	in	βF	pho (x)				39	39
								168
								170 (x)
159								169
								171 (x)
162	out	loop	phi (x)					
165	out	loop	phi (x)					
166	out	loop	phi (x)					
168	out	βG	Pho					39
								48
								158
							190	190
169	in	βG	pho (x)			205	205	205 (x)
								159 (x)
170								158 (x)
								190 (x)
171								159 (x)
189								52
								205 (x)
190	in	βH	pho (x)			48	48	48 (x)
							168	168
								170 (x)
191	in	βH			Small (x)			
192	in	βH	phi (x)	**NEG**	bulky	48	48	48 (x)
205	out	H2	pho (x)			169	169	169 (x)
								189 (x)
214	out	loop	pho					
220	out	βI	pho					52

Conserved positions based on the VAST alignment and related structural information: the position of the residue relative to the barrel; inside (in) or outside (out) and the corresponding secondary structure (2^nd^). The conserved properties (10/10 conservation) are also presented, i.e. hydrophobic (pho), hydrophilic (phi), charged (NEG for negative charge), aromatic (arom), bulky/small, as well as the conserved interaction partner (for 11/11, 10/10 and 9/10 lipocalins). (x) indicates the properties and interactions shared by 1QFT.

Interactions conserved for 9 proteins out of ten (9/10) are represented in Figure 2 and 3 and summarized in Table 1. Two clusters of hydrophobic interactions clearly appear. The internal cluster implicates residues on β-strands at the bottom of the barrel (Figure 3A). These residues can be seen as forming a hydrophobic belt. The internal cluster also includes H1. The external cluster involves residues on H2, strands β A, F, G and H and the C-terminal loop (Figure 2 and 3B). Globally, it appears that the net of interactions between strands β F, G and H is more developed than between the strands β A, B, C, D. Interaction 159–171 is not strictly conserved; for 1A3Y it is hydrophilic instead of hydrophobic.

The distance between residues may account for the reason why some interactions are not conserved for all 10 lipocalins. This is the case for interaction 159–171 in 1EXS, 115–131 in 1A3Y and 158–168 in 1NGL. Noticeably, interaction 168-48 is not conserved for 1NGL, because the orientation of Trp 48 is different than for other structures. Other interactions are not fully conserved because one residue is of a different nature than the other. This is the case for interactions 159–169, 189–205 and 52–189 that are not conserved for all ten proteins because residue 159 (Ser) in 1A3Y and 189 (Ser) in 1EXS are hydrophilic. This is also the case for interactions 158–170 and 170–190 in 1AQB. Some interactions that are not conserved are nevertheless compensated. For example, the hydrophobic interaction 80–93 is not conserved for 1PEE because of the hydrophilic nature of position 93 (Glu). However, this residue interacts with the hydroxyl groups of Tyr 39, 131 and 154, stabilizing the protein [23]. It is worth noting that the interaction 91–115 is not conserved for Nitrophorin 2 (1PEE), but is well conserved in its close homologue, Nitrophorin 4 [PDB: 1D2U] [23]. The interaction 170–190 is not conserved for 1AQB because of the hydrophilic nature of residue 170 (Gln). However, the lack of interaction 170–190 seems to be compensated for the external disulfide bond between Cys 173 and 182, linking β-G and β-H. The hydrophobic interactions 189–205 and 52–189 are not conserved owing to the hydrophilic nature of residue 189 in 1EXS. Nevertheless, Phe 205 interacts with both 52 and 189 and it appears to bridge these residues, the aromatic ring being in interaction with the Cβ of Ser (189). In addition, the interaction 52–220 is absent in 1NGL but compensated by another interacting pair, 219-54.

#### Interactions for 1QFT

1QFT is an outlier lipocalin with low similarity with the other lipocalins. It lies apart from the family since it does not share the conserved regions (SCRs) of the family, it binds hydrophilic ligands and H1 is in α-conformation. 1QFT can accommodate two hydrophilic ligands (histamine) in its cavity, one near H1 (L site) and the other near the mouth of the barrel (H site). The L site contains two charged residues, Asp 42 and Asp 168. Residue 168 (Asp) also interacts with Ser 38 and Ser 161. The H site contains four charged residues Asp 58, Glu 113, Asp 156 and Glu 188. Three aromatic residues are in close contact with the cycle of histamine. These are Trp 69 and Phe 154, which are parallel to the cycle and Tyr 133 that is perpendicular. When the interactions conserved for all 11 structures (10 lipocalins + 1QFT) are studied, it appears that only four are conserved; three located at the junctions between the two sets of strands (A-B-C-D and E-F-G-H) and one linking strand βG to H2 (Figure 2). One interaction implicates an aromatic (48) and a hydrophilic (192) residue (interacting through their hydrophobic parts), the other interactions are hydrophobic (48–190, 115–133, 169–205).

Conserved interactions identified at 90% (9/10 study) for lipocalins and shared by 1QFT (9 lipocalins + 1QFT) are shown in Table 1 and Figure 2C. Several interactions conserved for the 10/10 lipocalins study are not conserved in 1QFT. In the case of interaction 48–80, the lack of conservation is due to the distance. For interaction 91–131, this is due to the orientation of residue 131 towards the ligand. For interactions involving residues 156 and 168, their hydrophilic nature is responsible for the non-conservation; they both interact with histamine. Interactions 52–220 and 52–189 are not conserved, owing to the hydrophilic nature of residue 52, but actually Lys (52) appears to interact with Leu (189) through its hydrophobic part. Compensating interactions exist, residue 51 (Val) interacts with Leu 189 and Asp 221 interacts with Lys 52. Other non-conserved interactions in 1QFT may be partially compensated by proximate interactions. Interaction 48–78 may partially compensate for the loss of interaction 48–80, 95–133 may compensate for 91–131. Interactions 131–158, 161–168 and 170–190 may compensate respectively those between 131–156, 156–168 and 168–170.

### Helix 1

In the 1XKI and 1A3Y structures, residues close to or belonging to the N-ter part of the 3_10 _helix (H1) are absent or not well resolved. For that reason, the conservation was studied in the N-terminal region for 7 (7/8 study) and 8 (8/8 study) structures out of 10.

#### Conservation of the positions in the alignment

When the N-ter sequences of 1XKI and 1A3Y are considered (not in the structure but by aligning the sequences), positions 35 (phi), 38 (phi) and 39 (pho) have conserved properties in the H1 region. By comparing the proteins without 1XKI and 1A3Y, positions 26 (phi) and 34 (pho) are further conserved (position 26 does not exist in 1XKI and 1A3Y). For 1QFT, positions 38 and 39 are conserved: position 42 is not a Gly but preserves a small volume.

#### Interactions study of helix 1 for lipocalins

Conserved interactions in H1 for 8/8 and 7/8 structures are represented in Figure 2B and summarized in Table 2. The interactions are all hydrophobic except for that between residues 35 and 38 (hydrophilic). Three interactions are 100% conserved and three others are further conserved for 7/8 structures. Figure 2B illustrates the importance of the central residue (39) in the stabilization of the bottom of the barrel. It interacts with five out of eight strands (β-A, B, C, F and G) for 7/8 structures. The distance between the residues of interaction 39–168 (for 1NGL) and 39–48 (for 1EXS) explains why they do not appear in the 8/8 conservation study. Interaction 39–91 is missing in the 8/8 study because in 1PEE the side chain of residue 40 (Phe) is inserted between the two residues.

**Table 2:** Conserved properties and interactions for Helix 1.

Position	in/out.	Conserved properties		Conserved Interactions		
				9/10	8/8	7/8

26	out	phi	8/10			
34	in	pho	8/10			
35	out	phi	10/10		38	38
38	out	phi	10/10		35	35
39	in	pho	10/10			48
39				80	80	80
39						91
39				158	158	158
39				168		168

Positions in H1 that are conserved and related structural information i.e. the position of the residue relative to the barrel; inside (in) or outside (out). The conserved properties (10/10 and 8/10 conservation) are also presented (see table 1 for details), as well as the conserved interaction partner (for 9/10, 8/8 and 7/8 lipocalins).

#### Interactions of H1 for 1QFT

Due to the orientation and the α-conformation of H1 in 1QFT, residue 39 is not central as it is for the other lipocalins and thus is not involved in conserved interactions. Hence, the way by which α-helix 1 interacts with the barrel has been studied separately, as shown in Figure 2D. As for the other lipocalins, strands β-A, B, C, F and G interact with H1. The interactions involving residue 39 in the other lipocalins are balanced in 1QFT by those between residue 41 (Ala) and 48 (Tyr), 42 (Asp) and 80 (Ala), 39 (Leu) and 91 (Ile), 35 (Ala) and 158 (Ile), and 38 (Ser) and 168 (Asp). Furthermore, residue 37 (Lys) makes an electrostatic bond with residue 165 (Asp), as does residue 34 (Asp) with 125 (Arg) of the βD-βE loop. The interaction between residues 35 and 38 is replaced by that between 34 and 37(Asp-Lys).

#### Positions with a conserved nature not showing interaction conservation

It was noticed that several positions with conserved properties in the alignment are not implicated in conserved interactions in the 9/10 study. These are positions 26 (phi), 34 (pho), 38 (phi), 49 (bulky and hydrophobic), 74 (phi), 110 (phi), 162 (phi), 165 (phi), 166 (phi) and 214 (pho) and the positions with a conserved small volume (42, 55, 191). It was analyzed whether these positions were implicated in less conserved interactions. The positions with a conserved small volume are not implicated in conserved interactions. The hydrophilic positions 26 and 74 do not participate in highly conserved interactions. For position 34, a conserved interaction was found with residue 39 for 6/10 structures. Furthermore, position 34 is involved in a hydrophobic interaction with βE (residue 129 and/or 131) or βF-βG loop (residue 163) for 6/10 structures. It should be noted that for 1XKI and 1A3Y, position 34 is not hydrophobic. This may reduce the stability in this region and explain the difficulties in solving the conformation of H1.

Position 49 is implicated in a conserved hydrophobic interaction (for 7/10 structures) with position 194 (loop βG-H2), it is also conserved in 1QFT (not conserved in 1I4U, 1NGL and 1A3Y because these residues are too far apart from each other). Residue 110 (Pho) is interacting with 94 for 7/10 structures and with 96 for 6/10 structures. For 1QFT, position 110 is interacting with 96. Positions 162, 165, 166 are part of the γ-turn between βF and βG and are also conserved for 1QFT. Residue 162 interacts with 166 for all structures except for 1XKI. This interaction is not seen in the 9/10 conservation study, due to the restraint that residues must be separated by at least two residues to be considered in interaction. For 1QFT, it appears that residue 162 cannot make a H-bond with 166 owing to the presence of a disulfide bond linking βG to H2. Position 165 makes a hydrophilic, but not well conserved interaction (4/10 structures) with 38. The corresponding interaction for 1QFT is 37–165. This interaction might play an important role in the folding despite its low conservation, because both positions are well conserved. Position 214 interacts with residue 54 (5/10; pho), 187 (5/10; pho) and 209 (6/10; pho), reinforcing the external cluster.

### Homology modeling for LIR2

**Figure 4:**
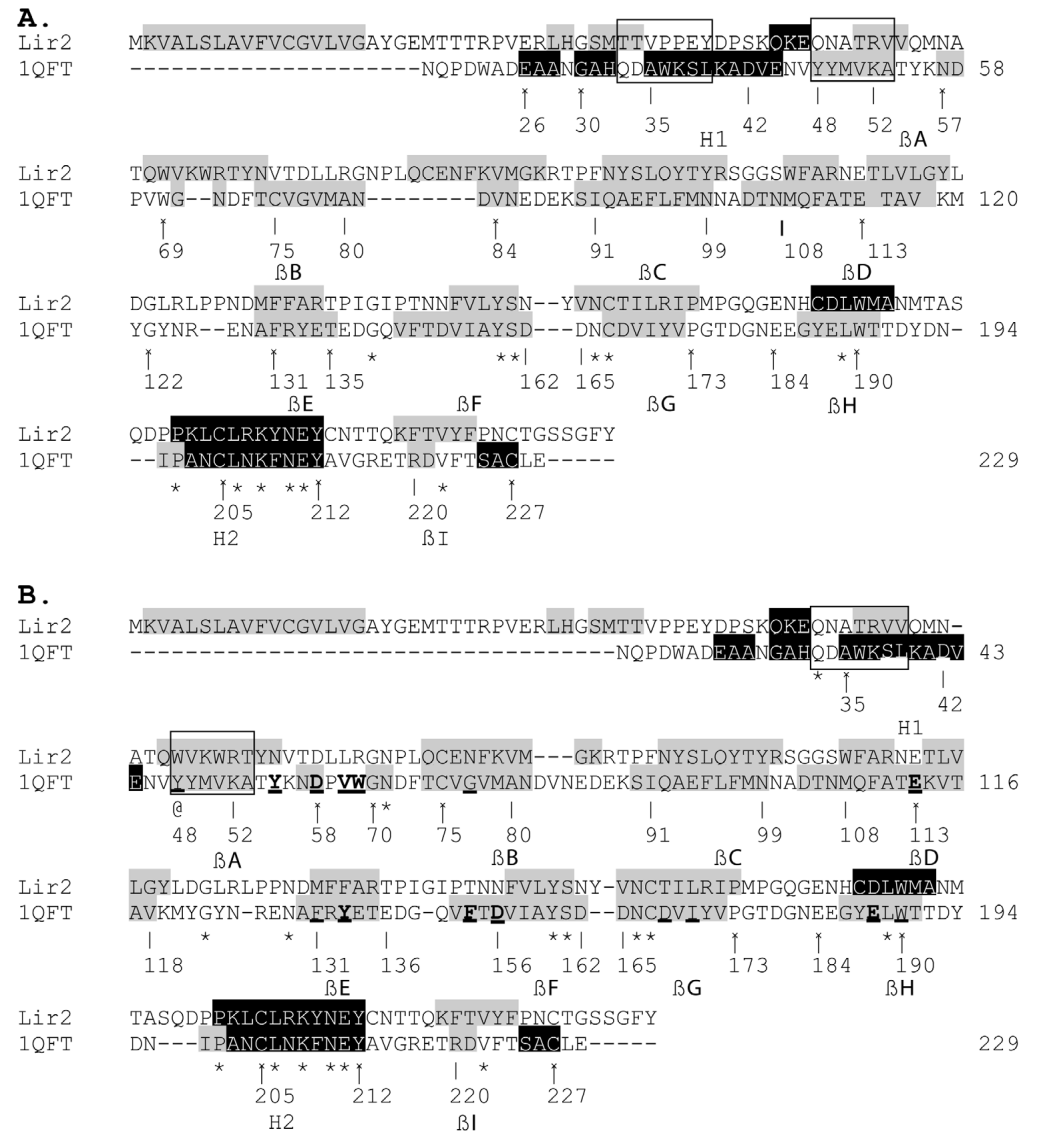
**ClustalW (A) and refined (B) alignment of LIR2 and 1QFT**. The secondary structure of 1QFT and the prediction for LIR2 (PROF) are shown. helix is in black (text in white) and β in gray. The numbering of 1QFT refers to that of Figure 1. Positions at the extremities of the secondary structure elements are numbered to facilitate the reading. Residues interacting with histamine in the structures of 1QFT are underlined. Those belonging to the H site are bold. Boxes indicate examples of realigned regions.

LIR2 is a protein from *Ixodes ricinus*. PSI-BLAST was used to scan the PDB to find a homologous protein [24]. The only structure found after 4 iterations with an E value inferior to the threshold was that of RaHBP2 (1QFT). LIR2 has an identity around 15% with 1QFT and no lipocalin recognition motifs. The ClustalW alignment between LIR2 and 1QFT is shown in Figure 4. As for the PSI-BLAST alignment, some aberrations are noticed. The secondary structure of LIR 2 (predicted with the PROF method [25]) does not correspond to that of 1QFT in the N-ter region. Furthermore, the region of LIR2 corresponding to H1 (in 1QFT) contains three prolines, that do not favor the helical conformation. In the region corresponding to βA, position 48 (referring to the lipocalin alignment of Figure 1) does not correspond to an aromatic amino acid in LIR2. This residue is aromatic for all lipocalins including 1QFT; several mutational studies have notably demonstrated the importance of that residue in the lipocalin structure stability [26-28]. Position 49 is not bulky and hydrophobic as in the other lipocalins. The region corresponding to βB is not predicted as β. Position 80 corresponds to an Arg, while being a hydrophobic residue in lipocalins. The cysteine from βB, implicated in a disulfide bridge between the C-ter part and βB in 1QFT, is also not conserved.

#### Modification of the alignment

To alleviate those misalignments, the alignment has been manually modified taking into account the prediction of secondary structures, the conserved interactions and positions for lipocalins and the cysteines implicated in disulfide bridges for 1QFT.

##### H1

To align H1 the conserved interaction between residues 34 and 37 is used (see the window on Figure 4). The corresponding residues for lipocalins and 1QFT are both hydrophilic. Positions 35 and 39 are also used: for 1QFT, residue 35 (Ala) is small allowing to decrease the steric constraints between H1 and, βD and βE. A bulkier residue at that position would interfere with the interaction with βD-βE. Residue 39 makes a hydrophobic interaction with 91. In the modified alignment, residues 34, 35, 37 and 39 now correspond respectively to Asn, Ala, Arg and Val.

For the N-ter region, the secondary structure and information about disulfide bonds did not aid the alignment since, it is predicted as β-strand by PROF for LIR2 but with a low reliability and is predicted as an α-helix by NPSA (data not shown).

##### βA

Residues 48 and 49 are implicated in conserved interactions: position 48 is a conserved Trp interacting with 80, 190 and 192 and 49 is a bulky hydrophobic residue interacting with 194. They both were used to align βA together with secondary structure predictions (see the window on Figure 4). The latter helps to obtain a global alignment but is not sufficient to avoid ambiguity, since it can be aligned in different ways. Adding restraints for residues 48 and 49 permit the ambiguity to be eliminated. In the modified alignment, they respectively correspond to Trp and Val for LIR2. Residues 80, 190, 192 and 194 are hydrophobic and susceptible to interactions with 48 and 49.

##### βB

To align βB, positions 75 and 80 were used together with secondary structure predictions. Residue 75 is a conserved Cys for arthropod lipocalins and residue 80 makes a conserved hydrophobic interaction with 93. Secondary structure predictions permit a global alignment to be obtained and residues 75 and 80 eliminate ambiguity.

##### βC and βD

Strands βC and βD were not realigned as the secondary structure predictions are in good correspondence with that of 1QFT and as positions 91, 93 and 115 are conserved hydrophobic residues. The corresponding residues for LIR2 are Phe, Tyr and Leu respectively. Residues 91 and 115 make conserved interactions with 80, 115 and 131. They all correspond to hydrophobic residues in the modified alignment.

##### βE

In strand βE, the secondary structure predictions are in good correspondence with that of 1QFT. Furthermore, residues 131 and 133, both implicated in conserved hydrophobic interactions, are conserved residues in the ClustalW alignment. Nevertheless, an uncertainty remains about the alignment of that region. Indeed, if a gap is suppressed in N-ter to βE, the secondary structures are still in good correspondence and residues 131 and 133 are still hydrophobic residues.

To determine the correct alignment, position 129 is used. In 1QFT, Asn 129 makes two H-bonds with the NH and CO groups of the backbone of the twisted βD-βE loop. This loop is of equivalent length in 1QFT and LIR2, and is longer than for other lipocalins. In the case where a gap is suppressed in N-ter, residue 129 corresponds to Asn for LIR2. For this reason, the latter was chosen. In that case, residues 131 and 133 correspond respectively to Met and Phe.

##### βF, βG and βH

Strands βF, βG and βH were not realigned as the secondary structures are in good correspondence and as residues 158, 167, 169, 170, 189 and 190 (respectively Val, Cys, Ile, Leu, Leu and Trp in LIR2) are potentially able to make the conserved interactions.

##### H2 and βI

H2 and βI were not realigned, as positions 205 and 227 are both Cys, as in 1QFT. In the latter, two disulfide bridges are present. One joins the C-ter part to βB and is conserved for arthropod lipocalins (75–227; 1QFT, 1PEE, 1I4U). The other bridges H2 to βG (167–205; also present for 1I4U). The corresponding Cys of LIR2 are conserved; furthermore, LIR2 possesses two supplementary cysteines that could form a disulfide bridge between H2 and βH.

In the modified alignment, almost all residues in the hydrophobic internal cluster of LIR2 are conserved, only residues 156 (Asn) and 168 (Thr) are hydrophilic, as for 1QFT. In the external cluster, positions 52 and 171 are Arg. Despite their hydrophilic nature, they are able to make hydrophobic interaction through their hydrophobic tail [29]. The sequence corresponding to loop βF-βG in LIR2 is similar to the SCR2 motif of the lipocalins. Residues Thr-Asp-Tyr in 1AQB are equivalent to Ser-Asn-Tyr in LIR 2 [3].

The fairly good correspondence between the secondary structures of 1QFT and LIR2, combined with the conservation of the residues implicated in the two conserved hydrophobic clusters and the conservation of the Cys involved in disulfide bridges in 1QFT, lend support that LIR2 belongs to the lipocalin family.

##### 3D model

A 3D model was constructed using the refined alignment and the 1QFT structure as template. Modeller was used to build the model [30]. Its stereochemical validity was checked with the Procheck algorithm [31]. Only one residue is in the disallowed phi/psi region of the Ramachandran plot. Three others, located in loops, are in generously allowed region. In the model, it is noted that the two cysteines located on βH and H2, have no correspondence in 1QFT and are facing each other (residues 187 and 213 on Figure 3D). The distance between the Cα of the two residues is 6 Å, compatible with a disulfide bridge. For that reason, a model where Cys 187 and Cys 213 were restrained to form a disulfide bridge was calculated. This model is similar to that built without restraint (data not shown). In the model, all interactions conserved for lipocalins (9/10 study) are found for LIR2, except for that between residues equivalent to positions 48 and 80. As for 1QFT, residue 48 interacts with 78. Neither interactions with residue 91 (Phe) are conserved owing to the orientation of its side chain, which points outside. Interactions involving residues 156 and 168 are not conserved in LIR2 because of their hydrophilic nature. As for 1QFT, the residue equivalent to 52 (Arg) interacts through its hydrophobic tail with 189 (Leu) and interacts with 221 (Thr).

#### Experimental measurement of the secondary structure

FTIR measurements permitted the determination of the secondary structure of LIR2. The FTIR spectrum presents a maximum at 1632 cm^-1^, characteristic of β-structure (data not shown). After deconvolution, there is 22% of α-helix, 48% of β-strand, 17% of turns and 13% of coil. This is typical of lipocalins, notably 1QFT that has 19% α-helix, 43% of β-strand, 24% of turns and 13% of coil, as determined on the RX structure.

#### Prediction of ligand binding

The analysis of the internal cavity of LIR2 reveals that the bottom of the barrel is more hydrophobic than for 1QFT and that the upper part contains almost all the hydrophilic residues of the cavity. As shown in Figure 4, the hydrophobic residues in the bottom of the barrel are conserved between LIR2 and 1QFT; i.e. Trp (48), Phe (78), Val (80), Tyr (93), Leu (115), Met (131), Phe (133), Val (158), Leu (170) and Trp (190).

When comparing the residues of the L site, that participate in the binding of the histamine in 1QFT, to the corresponding residues in LIR2, it appears that the negative residues Asp 42 and 168 (see Figure 4) are not conserved. In 1QFT, these have been shown to interact with histamine. The corresponding residues in LIR2 are Asn (42) and Thr (168). As no negative residue is conserved in the L site for LIR2, no binding of histamine is predicted for that site.

Concerning the H site in LIR2, the negative residues are pretty well conserved; only residue 156 (Asn) is not. However, LIR2 contains a positive residue (Lys 50) in the cavity and two others (Arg 69 and 111) that are susceptible to belong to the ligand-binding pocket; these would repulse for histamine binding. Furthermore, the aromatic residues (Trp 69 and Phe 154) that are parallel to the cycle of histamine in 1QFT are not conserved in LIR2 (respectively Arg and Thr). For RaHBP1 (a close homologue to RaHBP2), such a substitution (Phe 154 is substituted by Leu) causes a significant decrease in affinity for histamine [6]. Furthermore, in the loops surrounding the entry of the H site, the ratio of negative to positive residues is 7/1 for 1QFT and 2/3 for LIR2. In 1QFT, the presence of these negative residues in the loops were proposed to contribute to the attraction of histamine to the binding site [6]. For the H site, despite the fact that most of the negative histamine-binding residues are conserved, the absence of the aromatic residues and of one negative residue should hinder high affinity binding of histamine for LIR2.

#### Experimental determination of the affinity of LIR2 to histamine

**Figure 5:**
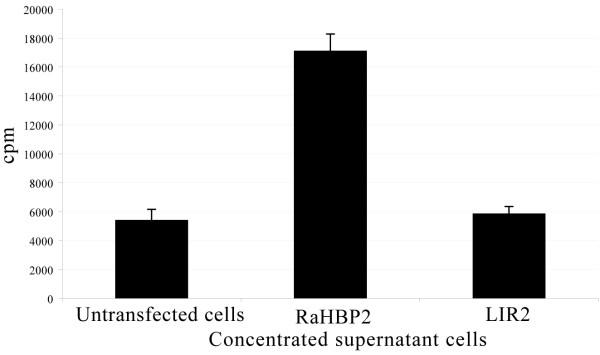
**Binding assay of LIR2 with histamine**. Binding assay was performed with 40 μl of concentrated 293T supernatant cell culture. The negative control used was a 10 time concentrated free-serum medium of untransfected cells. The supernatants were incubated with 100 nM ^3^H-histamine for 2 hours at 37°C. Protein precipitation with polyethylene glycol 8000 was used to separate bound from free histamine.

LIR2 and RaHBP2 were expressed in 293T free-serum cell medium. The ability of LIR2 to bind histamine was tested by incubating concentrated supernatant cells containing LIR2 with ^3^H-histamine. RaHBP2 was used as positive control, and a concentrated supernatant of untransfected cells used as negative control. These binding assays show high affinity for RaHBP2, and no affinity for LIR2 (similar cpm value to supernatant of untransfected cells) confirming that LIR2 is unable to bind histamine, as predicted from the model (Figure 5).

## Discussion

The aim of the present work is to provide information to help the construction of 3D models for the weakly related proteins of the lipocalin family. The members of this family have a wide variety of functions and are hence of biological importance. The identity between lipocalins can fall below 10%. Building a 3D model by homology modeling for proteins having an identity below the 25–30% cut-off is quite risky and requires that the selection of the template and the alignment with the target be further validated. This can be done by comparing predictions of secondary structures, accessibility to the solvent and patterns of hydrophobic and peculiar residues. In this work, information about the structural core of lipocalins was extracted and used to build a 3D model for LIR2, a protein from the tick *Ixodes ricinus*. For that purpose, a set of lipocalin structures was analyzed and conserved properties were identified. To capture the widest diversity it was tried to find a structure for each clade identified in the phylogenic study of lipocalins (Ganfornina et al., 2000). Nine structures were collected. Nitrophorin 2 [PDB: 1PEE] and RaHBP2 [PDB: 1QFT] were included into the study. The latter was studied separately owing to its uncommon α-conformation of the first helix and to its hydrophilic binding sites. The lipocalins were structurally aligned with the VAST method.

### Conserved positions of the alignment

To analyze the conserved properties of the alignment, the amino acids were classified as hydrophobic, hydrophilic, aromatic, charged, bulky or small. For the 10 lipocalins, having a mean length of 170 amino acids, only 25 positions are conserved, two are kept strictly identical (Gly (42) and Trp (48) from SCR1) and one is negative (192 from SCR3). The ratio of conserved hydrophobic versus hydrophilic positions is nearly three to one. All conserved hydrophobic positions have a solvent accessible surface less than 30%. The size of the residues is less conserved than their hydrophobicity. Only 7 positions are conserved in terms of size; however this is not unusual [32].

**The side chain-side chain interactions **were studied for each structure. Interactions were divided into four classes depending on the nature of the residues implicated, i.e. hydrophobic, hydrophilic, charged or aromatic. At first, RaHBP2 was not considered in the analysis and the conservation of interactions for 10/10 and 9/10 structures were analyzed. In both studies, no conserved electrostatic or aromatic interactions were found and mostly all conserved interactions were hydrophobic. The pattern of hydrophobic interactions suggests the existence of two clusters, one internal to the barrel and one external. The internal cluster is composed by residues 39, 48, 80, 91, 93, 115, 131, 133, 156, 158, 168, 170, 190 and 192 (Figure 3A), and the external by residues 52, 159, 169, 171, 189, 205 and 220 (Figure 3B), when considering the 9/10 study.

**In the internal cluster, **the β strands are linked by 14 hydrophobic interactions (Figures 2A and 3A), forming what can be seen as a hydrophobic belt. A similar belt was detected in the 10 β-strand barrel of the lipid-binding protein family [33,34]. This belt is linked to the central residue (39) of H1 by 5 interactions, coming from 5 different strands (Figure 2B). The central residue hence appears important in the structural core of lipocalins. The helix is further stabilized by an internal hydrophilic interaction. Among the conserved positions in the alignment and not appearing in the conserved interactions (9/10 and 7/10 study; Figure 1) are positions 34, 38, and 165. They are involved in less conserved interactions between the barrel and H1.

**The external cluster **implicates helix 2 and strands β-A, β-F, β-G, β-H as well as the C-terminal region; six interactions are conserved (Figures 2A and 3B). There is also an additional hydrophilic interaction (162–166) that stabilizes the βF-βG loop (SCR2).

It should be noted that all conserved hydrophobic positions in the alignment are implicated in conserved interactions, except positions 49 and 214, for which interactions are conserved for a fewer number of structures. These last two positions belong to the external cluster. Among the 6 conserved hydrophilic positions in the alignment, three are implicated in SCR3 and one (110) seems to be involved in the stabilization of the interaction between strands βB and βC on the external surface of the barrel.

Our results were compared to those of Ragona and col. [20] who have identified by NMR the interacting residues in partially folded bovine β-lactoglobulin at pH 2. These residues located in the cavity of the barrel correspond to positions 39, 48, 77, 80, 91, 131, 156, 158, 168, 170 and 190 in our alignment. Residues 93, 95, 115, 133 could not be unambiguously detected by NMR, but they were assigned by the authors to the internal cluster using the X-ray structure. All these residues are in good correspondence with those identified in the present study. The interacting residues in the external cluster (detected by NMR) correspond to positions 52, 167, 169, 204, 205, 208. Residues 51, 77 were furthermore assigned to this cluster using the X-ray data. Residue 77 was not detected as making conserved interaction in our study. This is due to the presence of a beta bulge in the 77–80 region. This bulge is present in our study for β-lactoglobulin [PDB: 1EXS], the odorant binding lipocalin from nasal mucosa of pig [PDB: 1A3Y] and the mouse major urinary protein [PDB: 1DF3]. Residues 204 and 208 respectively interact with residues 167 and 169 in the NMR study. In the present work, residues 204 and 167 do not appear to interact owing to the orientation of H2 towards the barrel that differs for β-lactoglobulin. Residues 208 and 169 were not detected to be involved in a strictly conserved interaction. In effect they show interaction for 6/10 structures and two structures have an Arg at position 208 interacting through its hydrophobic region with residue 169. Likewise, residue 208 (Lys) of RaHBP2 is also interacting with 169 through its hydrophobic region.

It was suggested that β-lactoglobulin at acidic pH is in a molten globule state, similarly to the retinol binding protein [26]. Since the residues implicated in interactions in the β-lactoglobulin molten globule correspond well with those conserved for native lipocalin structures, it supports the hypothesis that residues essential in the native structure of the lipocalins are also important for the folding, as suggested by Ragona et al. [35]. Clarke et al. reached a similar conclusion for the immunoglobulin-like proteins, a highly diverse protein family with no conservation of function and little or no sequence identity [16].

Greene et al. have studied the evolutionarily conserved residues (ECR) in 32 lipocalins [19]. Many of those residues are hydrophobic and equivalent to those highlighted in this work (residues 34, 39, 42, 48, 49, 52, 54, 129, 131, 158, 161, 162, 163, 167, 189, 191, 192, 200, 203 and 205 in our alignment). However, no residues from βB, βC, βD are found conserved by Greene et al. Even if fewer interactions are conserved for these strands, our study and that of Ragona *et al.*[20] clearly suggest that some residues of these strands also play a role in the hydrophobic internal cluster, closing the belt. This discrepancy could be due to the fact that our alignment is based on the structures and not on the sequences alone. Some other residues, such as 161 and 163 belonging to the βF-G loop (SCR2) are described as ECR, but are not found as conserved in this study. This is because outlier lipocalins were included in the alignment. Since residues 133 and 190 are implicated in conserved interactions for 11/11 structures it is surprising not to see them in the ECRs. Again this could be due to the way lipocalins were aligned.

### RaHBP2

In our alignment, RaHBP2 [PDB: 1QFT] was added. The latter is an outlier lipocalin with a low similarity to the other lipocalins. It lies apart from the family since it does not share the conserved regions of the family, binds a hydrophilic ligand and its H1 is in α-conformation. When considering RaHBP2 in the conservation analysis, it comes out that only four interactions are conserved for the 11 lipocalins, three in the internal cluster and one in the external. The interactions in the barrel link the two sheets (ABCD and EFGH) together. When comparing the interactions conserved for the 9/10 lipocalins to those of RaHBP2, it appears that the belt is not fully conserved owing to the hydrophilic nature of two residues (156 and 168) and to the orientation of residue 131, involved in the binding with histamine. The interaction 48–80 is not present: due to the α-conformation of H1, residue 48 moves away from residue 80. Nevertheless, there are neighboring interactions than can compensate these lacks. H1 of RaHBP2 has no central residue equivalent to residue (39) interacting with strands βA, βB, βC, βF and βG, while still interacting with those strands through different residues.

Thus, even though H1 has a different conformation and though there are two hydrophilic binding sites, the hydrophobic internal cluster of RaHBP2 is fairly well conserved. The external cluster of RaHBP2 is conserved except for interactions 52–189 and 52–220 because of the hydrophilic nature of residue 52, but a careful analysis reveals that residue 52 interacts through its hydrophobic moiety with residue 189. For RaHBP2 (as for α-crustacyanin [PDB: 1I4U]), a disulfide bond bridges βG to H2.

### Modeling of LIR2

To determine the homology and build a 3D model for LIR 2, a protein with only 15% identity with RaHBP2 [PDB: 1QFT], information was combined from the analyses of the structural core of lipocalins, the positions of cysteines implicated in disulfide bridges in RaHBP2 and the secondary structure. In a first approach, alignment between LIR2 and RaHBP2, was carried out by ClustalW. The alignment showed inconsistencies in the Cys bonding pattern, the secondary structures and the conserved hydrophobic residues. It was corrected for H1 and strands βA, βB and βE. The information obtained from the comparative analysis enabled the alignment to be improved. Due to the low similarity between the two sequences, the secondary structure prediction of LIR2 and the cysteine bridge conserved among arthropod lipocalins did not provide enough information to obtain an unambiguous alignment. This holds true for PSI-BLAST alignment (not shown). The information from conserved interactions has permitted to obtain a coherent alignment. It is important to note that the analysis of the structural core is not aimed to perform better than PSI-BLAST (or ClustaW), but rather to eliminate ambiguities and assess the alignment obtained by those methods.

The corrected alignment enabled the building of a 3D model for LIR2. The model shows a potential disulfide bridge, not present in RaHBP2, supporting both the assignment of the fold and the alignment (Figure 3D). This is further supported on one hand by the FTIR measurements that indicate a secondary structure compatible with the lipocalin fold and on the other hand by the conservation in LIR2 of most of the conserved hydrophobic interactions. Despite its homology with RaHBP2, the analysis of the model of LIR2 does not suggest binding to histamine, as confirmed experimentally. A more detailed study of the cavity should further help to understand the nature of its natural ligand.

## Conclusion

The lipocalins are part of a protein super-family with a low level of pairwise similarity, making homology modeling a difficult task. In this study, it was shown that the determination of the residues implicated in the hydrophobic core of lipocalins, by analyzing the conserved interactions, enabled to assess the assignment of a lipocalin-like protein and to improve the "classical" alignment in ambiguous regions. Information obtained from that study should help modeling other lipocalin-like proteins. This study could be applied to other protein families with low pairwise similarity, such as the structurally related fatty acid binding proteins or avidins.

## Methods

### Computational methods

#### Lipocalin analysis

To study the lipocalin family a bank of structurally aligned lipocalins with low similarity was gathered. For this purpose, it was tried to obtain a structure for each of the clades identified in the phylogenic analysis of Ganfornina and col. [36]. A 3D structure was found in the Protein Data Bank (PDB) for nine out of 13 clades. Since nitrophorin [23], from *Rhodnius prolixus *and RaHBP2 from *Rhipicephalus appendiculatus *were not considered in the phylogenetic tree owing to their low similarity, they were added in the bank. The structural alignment was generated by the VAST algorithm [37] and includes the odorant binding lipocalin from nasal mucosa of pig [PDB: 1A3Y], the retinol binding lipocalin of pig [PDB: 1AQB], the mouse major urinary protein [PDB: 1DF3], the beta-lactoglobulin of pig [PDB: 1EXS], α-crustacyanin [PDB: 1I4U], Human Complement Protein C8 γ [PDB: 1LF7], the human neutrophil gelatinase-associated lipocalin [PDB: 1NGL], nitrophorin 2 from *Rhodnius prolixus *[PDB: 1PEE], the *Rhipicephalus appendiculatus *histamine binding lipocalin 2 [PDB: 1QFT], the bacterial outer membrane lipoprotein blc [PDB: 1QWD] and the human tear lipocalin [PDB: 1XKI]. The retinol-binding protein (1AQB) is one of the best characterized lipocalin. Consequently, it was used as reference for the calculation of the deviation between the structures. The RMSD between 1AQB and the other structures is between 2 and 3 Å. For some structures, the natural sequence was not conserved thoroughly; some substitutions were introduced. For the human tear lipocalin, residue 161 is naturally a Cys but is a Ser in the structure 1XKI. In 1QWD, some residues have been mutated in the region before H1. In 1I4U, residue 94 (Lys) has been replaced by Glu [βC, facing outside]. This could destabilize the electrostatic interaction with Glu 79. Residue 88 in 1LF7A (Cys) has been replaced by Gly [loop between βB and βC]. In 1DF3 residue 207 (Lys) has been replaced for Gln [H2, facing exterior]. Furthermore, for 1XKI several loops are lacking in the structure, as well as H1 and the C-ter part. For 1A3Y, H1 is missing and for 1LF7 the omega loop is not present.

#### Study of the interactions

The interactions were computed from PDB files with the PEX software[22]. Previously, the PDB files corresponding to the structures were renumbered, so that spatially equivalent residues (i.e. having the same position in the structural alignment) have the same number. This procedure considers that amino acids interact when the center to center distance between their closest atoms is less than 4.5 Å. Residues which interact must be separated by at least by two residues in the sequence. The accessible surface area (ASA) was calculated using the method of Shrake and Rupley [38]. To be considered accessible (or inaccessible) to the solvent a residue has to have an ASA more (or less) to 30% of its total surface.

#### Modeling of LIR2

The PROF prediction of secondary structure was obtained through the PredictProtein server [25,39]). The ClustalW algorithm was used to generate the non-refined alignment between LIR2 and the sequence of 1QFT [40]. The 3D model of LIR2, comprising residues 22 to 196, was generated by Modeller [30], using a refined alignment. The model was afterwards evaluated by Procheck [31].

### Experimental methods

#### Sequencing LIR2

mRNAs from salivary glands of 30 engorged females of *Ixodes ricinus *were extracted using the Micro-FastTrack 2.0 mRNA Isolation Kit (Invitrogen, Carlsbad, USA). The complete cDNA sequence of LIR2 was recovered by RACE-PCR (Gene Racer Kit, Invitrogen) performed according to manufacturer's recommended procedure.

#### Characterizing LIR2

The molecular weight of LIR2 is of 24.2 kDa and its isoelectric point is of 8.97, as determined by "pepstats". The signal peptide is predicted by "SignalP" to be of 20 amino acids.

#### Plasmid construction, protein expression, and purification of LIR2 for FTIR measurements

The coding region of LIR2 was amplified by PCR (94°C for 30 s, 56°C for 30 s, 72°C for 1 min.; 30 cycles) with the ExTaq DNA Polymerase. The PCR product was ligated into the pCRII-TOPO vector and then excised with *EcoRI *and *SacI *sites that were added at the 5' end of the primers. The resulting DNA fragment was cloned into the pBlueBac4.5-V5-His (Invitrogen) in frame with the coding sequence of the V5 and His epitopes at the C-terminus. Recombinant baculoviruses were made by recombination between pBlueBac/LIR2 and Bac-N-Blue linear DNA virus (Invitrogen). Recombinant viruses were selected and amplified according to the manufacturer's instruction. SF9 cells were infected with a high-titer stock of recombinant baculovirus and were incubated for 72 hours at 27°C in Sf900 II Serum-Free Medium (Invitrogen). Recombinant LIR2 was recovered in 50 mM NaH_2_PO_4 _buffer (pH 7.5) containing 150 mM NaCl by using a Ni^2+ ^sepharose (Qiagen) column.

#### Expression of recombinant LIR2 and RaHBP2 for binding assays

Messenger RNAs were extracted either from salivary glands of 2-days engorged *Rhipicephalus appendiculatus *females or from salivary glands of 5-days engorged *Ixodes ricinus *females. The extraction was performed by using Micro-Fast Track 2.0 mRNA isolation kit (Invitrogen). The mRNA was reverse-transcribed into cDNA using SuperScript II Reverse Transcriptase (Invitrogen). The coding regions of both RaHBP2 and LIR2 genes were amplified by PCR (94°C for 30 seconds, 56°C for 30 seconds, 72°C for 1 minute; 30 cycles) by using the Takara ExTaq DNA polymerase. The PCR products were cloned into the pcDNA3.1/V5-His-TOPO (Invitrogen). The resulting recombinant plasmids were used to transfect 293T cells with FuGene 6 (Roche applied science, Indianapolis, USA); and the proteins were expressed in fusion with a V5 and a 6-HIS epitope tag. The proteins were produced in a serum- free medium. The supernatants were collected 72 hours after transfection, and concentrated 10 fold with an Amicon membrane (Cutoff of the membrane: 10000 NMWL). The supernatants were then dialyzed against PBS (20 mM NaH_2_PO_4_, 150 mM NaCl, pH 7.4) and ultracentrifugated at 140 000 g before used.

#### Binding assay with histamine

^3^H-histamine was purchased form GE Healthcare (Little Chalfont, Buckinghamshire, United Kingdom). Protein precipitation with polyethylene glycol 8000 to separate bound from free histamine followed the procedure described by Warlow and Bernard [41]. The radioactivity was counted in a Wallac 1409 scintillation counter.

#### Infrared spectroscopy (FTIR) measurements

Attenuated Total Reflection (ATR) infrared spectroscopy was used to determine the secondary structure of LIR2. Spectra were recorded at room temperature on a Brüker Equinox 55 equipped with a liquid nitrogen-cooled Mercury-Cadmium-Telluride (MCT) detector at a resolution of 2 cm^-1^, by averaging 512 scans. The internal reflection element was a germanium ATR plate (50 × 20 × 2 mm, Aldrich Chimica) with an aperture of 45° yielding 25 internal reflections. Reference spectra of a Germanium plate were automatically recorded after purge of 15 minutes with dry air and rationed against the recently run sample spectra. Seventy μg of protein was spread out on the plate and slowly dried under a stream of N_2_. The plate was sealed in a universal sample holder and rehydrated by flushing the holder with N_2 _saturated with D_2_O for 4 hours at room temperature.

#### Secondary structure determination

Vibrational bands, especially the amide I band (1600–1700 cm^-1^), are sensitive to the secondary structures of the proteins. The C=O vibration is representative of 80% of the amide I band. This band accounts for all the secondary structures that have different vibration values. The combination of resolution-enhancement methods with curve-fitting procedures allows quantitatively different secondary structures such as α-helix, β-sheets and unordered structures to be assigned. Each band was assigned according to the frequency of its maximum. The areas of all bands assigned to a given secondary structure were then summed and divided by the sum of all areas. This gives the relative ratio of each secondary structure. The bands are assigned as follows [42]: α-helix: 1662-1645 cm^-1^, β-sheets: 1689-1682 cm^-1 ^and 1637-1613 cm^-1^, random 1645-1637 cm^-1^, β-turns: 1682-1662 cm^-1^. It should be noted that the proteins spread on the plate are deuterated to avoid an overlap of α-helix and random-coil structures, as previously described [42].

## Authors' contributions

AB conceived the study, participated in the design of the study, carried out the lipocalin structural analysis, the alignment of LIR2 and its 3D model, and drafted the manuscript.

CB participated in the design of the study and drafted the manuscript.

BJ participated in the design of the experimental part, carried out the sequencing, characterization, expression and binding assays for LIR2 drafted the experimental part of the manuscript.

VL participated in the design of the experimental part and revised the manuscript.

GE participated in the design of the experimental part and revised the manuscript.

BR participated in the design and revised the manuscript.

LL conceived the study, participated in the design of the study and revised the manuscript.

All authors read and approved the final manuscript.

## Supplementary Material

Additional file 1 **Identity table for the lipocalins aligned by the VAST method**. These include the structures of the odorant binding lipocalin from nasal mucosa of pig (1A3Y, [43]), the retinol binding lipocalin of pig (1AQB, [44]), the mouse major urinary protein (1DF3, [45]), the beta-lactoglobulin of pig (1EXS, [46]), α-crustacyanin (1I4U; [47]), Human Complement Protein C8 γ (1LF7, [48]), the human neutrophil gelatinase-associated lipocalin (1NGL, [49]), nitrophorin 2 from *Rhodnius prolixus *(1PEE, [23]), the *Rhipicephalus appendiculatus *histamine binding lipocalin 2 (1QFT, [50]), the bacterial outer membrane lipoprotein blc (1QWD, [51]) and the human tear lipocalin (1XKI, [52]). On the diagonal is the number of residues for each sequence, in the upper triangle the number of identical residues and in the lower triangle the percentage of sequence identity (identities/length of the shorter sequence). Click here for file
