# Supplementary material for: Distantly related lipocalins share two conserved clusters of hydrophobic residues: use in homology modeling
Source: BMC Struct Biol. 2008 Jan 11;8:1. doi: 10.1186/1472-6807-8-1 (PMC2254393; doi:10.1186/1472-6807-8-1)
Supplement: Additional file 1 — Identity table for the lipocalins aligned by the VAST method. These include the structures of the odorant binding lipocalin from nasal mucosa of pig (1A3Y, [43]), the retinol binding lipocalin of pig (1AQB, [44]), the mouse major urinary protein (1DF3, [45]), the beta-lactoglobulin of pig (1EXS, [46]), α-crustacyanin (1I4U; [47]), Human Complement Protein C8 γ (1LF7, [48]), the human neutrophil gelatinase-associated lipocalin (1NGL, [49]), nitrophorin 2 from Rhodnius prolixus (1PEE, [23]), the Rhipicephalus appendiculatus histamine binding lipocalin 2 (1QFT, [50]), the bacterial outer membrane lipoprotein blc (1QWD, [51]) and the human tear lipocalin (1XKI, [52]). On the diagonal is the number of residues for each sequence, in the upper triangle the number of identical residues and in the lower triangle the percentage of sequence identity (identities/length of the shorter sequence). [file 1472-6807-8-1-S1.doc]

|  | 1A3Y | 1AQB | 1DF3 | 1EXS | 1I4U | 1LF7 | 1NGL | 1PEE | 1QFT | 1QWD | 1XKI |
| --- | --- | --- | --- | --- | --- | --- | --- | --- | --- | --- | --- |
| 1A3Y | **149** | 13 | 41 | 21 | 12 | 21 | 14 | 18 | 14 | 8 | 20 |
| 1AQB | 9 | **175** | 23 | 26 | 25 | 33 | 24 | 23 | 13 | 30 | 24 |
| 1DF3 | **28** | 14 | **161** | 27 | 16 | 25 | 24 | 12 | 16 | 17 | 24 |
| 1EXS | 14 | 16 | 17 | **160** | 22 | 30 | 24 | 14 | 9 | 22 | 27 |
| 1I4U | 8 | 14 | 10 | 14 | **181** | 20 | 18 | 18 | 21 | 28 | 19 |
| 1LF7 | 14 | 20 | 16 | 19 | 12 | **164** | 27 | 11 | 11 | 22 | 25 |
| 1NGL | 9 | 14 | 15 | 15 | 10 | 16 | **179** | 20 | 8 | 24 | 16 |
| 1PEE | 12 | 13 | 7 | 9 | 10 | 7 | 11 | **180** | 14 | 24 | 14 |
| 1QFT | 9 | 8 | 10 | 6 | 12 | 7 | **5** | 8 | **171** | 12 | 13 |
| 1QWD | **5** | 18 | 11 | 14 | 17 | 13 | 14 | 14 | 7 | **167** | 18 |
| 1XKI | 14 | 17 | 17 | 19 | 13 | 17 | 11 | 10 | 9 | 13 | **144** |
